# Positive end-expiratory pressure optimization with forced oscillation technique reduces ventilator induced lung injury: a controlled experimental study in pigs with saline lavage lung injury

**DOI:** 10.1186/cc10236

**Published:** 2011-04-28

**Authors:** Peter Kostic, Emanuela Zannin, Marie Andersson Olerud, Pasquale P Pompilio, Göran Hedenstierna, Antonio Pedotti, Anders Larsson, Peter Frykholm, Raffaele L Dellaca

**Affiliations:** 1 Department of Surgical Sciences, Anaesthesia and Intensive Care, Uppsala University, S 751 85 Uppsala, Sweden; 2 Dipartimento di Bioingegneria, Politecnico di Milano University, P.zza Leonardo da Vinci 32, 20133 Milano, Italy; 3 Department of Medical Sciences, Clinical Physiology, Uppsala University, 751 85 Uppsala, Sweden

## Abstract

**Introduction:**

Protocols using high levels of positive end-expiratory pressure (PEEP) in combination with low tidal volumes have been shown to reduce mortality in patients with severe acute respiratory distress syndrome (ARDS). However, the optimal method for setting PEEP is yet to be defined. It has been shown that respiratory system reactance (Xrs), measured by the forced oscillation technique (FOT) at 5 Hz, may be used to identify the minimal PEEP level required to maintain lung recruitment. The aim of the present study was to evaluate if using Xrs for setting PEEP would improve lung mechanics and reduce lung injury compared to an oxygenation-based approach.

**Methods:**

17 pigs, in which acute lung injury (ALI) was induced by saline lavage, were studied. Animals were randomized into two groups: in the first PEEP was titrated according to Xrs (FOT group), in the control group PEEP was set according to the ARDSNet protocol (ARDSNet group). The duration of the trial was 12 hours. In both groups recruitment maneuvers (RM) were performed every 2 hours, increasing PEEP to 20 cmH_2_O. In the FOT group PEEP was titrated by monitoring Xrs while PEEP was reduced from 20 cmH_2_O in steps of 2 cmH_2_O. PEEP was considered optimal at the step before which Xrs started to decrease. Ventilatory parameters, lung mechanics, blood gases and hemodynamic parameters were recorded hourly. Lung injury was evaluated by histopathological analysis.

**Results:**

The PEEP levels set in the FOT group were significantly higher compared to those set in the ARDSNet group during the whole trial. These higher values of PEEP resulted in improved lung mechanics, reduced driving pressure, improved oxygenation, with a trend for higher PaCO_2 _and lower systemic and pulmonary pressure. After 12 hours of ventilation, histopathological analysis showed a significantly lower score of lung injury in the FOT group compared to the ARDSNet group.

**Conclusions:**

In a lavage model of lung injury a PEEP optimization strategy based on maximizing Xrs attenuated the signs of ventilator induced lung injury. The respiratory system reactance measured by FOT could thus be an important component in a strategy for delivering protective ventilation to patients with ARDS/acute lung injury.

## Introduction

Mechanical ventilation is a mainstay of intensive care for patients with acute lung injury (ALI) and the acute respiratory distress syndrome (ARDS). A ventilation strategy based on tidal volumes of 6 ml.kg^-1 ^and pre-defined positive end-expiratory pressure (PEEP) settings has been shown to reduce morbidity and mortality probably due to less ventilation-induced lung injury (VILI) [1-3]. Various protocols using higher levels of PEEP in combination with low tidal volumes (Vt) have also been shown to reduce mortality in patients with ARDS [4], which was corroborated in a recent meta-analysis [5,6]. Meanwhile, experimental studies have been designed to define the optimal PEEP level based on lung compliance or elastance recorded during a recruitment maneuver (RM) with decremental PEEP [7,8].

We have recently shown that respiratory system reactance (Xrs) obtained by the forced oscillation technique (FOT) at 5 Hz is more reliable than dynamic compliance for assessing lung collapse and the effects of lung RMs in a porcine ALI model [9,10]. Specifically, Xrs (and its derived variable C_X5_, the oscillatory compliance at 5 Hz) identifies the minimum PEEP level required to maintain lung recruitment with high sensitivity and specificity. The advantages of this non-invasive approach are that it can be easily integrated in mechanical ventilators, it is suitable for bedside continuous monitoring, and it can also be used in the presence of spontaneous breaths.

During long-term ventilatory treatments, the optimal PEEP level is likely to change with time due to the developing disease process as well as various interventions in the ICU. Hence, a strategy designed to reduce VILI should probably include repeated assessment of lung mechanics, with subsequent changes in the ventilator settings.

The aim of the present study was to evaluate the effects of repeated PEEP optimization based on Xrs on oxygenation, lung mechanics, and histologic markers of lung injury, and compare them to the results obtained by applying the ARDSNet protocol based on oxygenation alone, in a porcine surfactant-depletion lung injury model over a 12-hour ventilation period. The hypothesis was that repeated PEEP optimization by FOT could improve lung mechanics and reduce VILI.

## Materials and methods

Seventeen healthy pigs (weight 26.6 ± 2.2 kg, Swedish mixed country breed) were studied at the Hedenstierna laboratory, Department of Surgical Sciences of the University Hospital of Uppsala, Sweden. The study was approved by the local animal ethics committee.

### Animal preparation

Anesthesia was induced by tiletamine 6 mg.kg^-1^, zolazepam 6 mg.kg^-1^, xylazine 2.2 mg.kg^-1 ^intramuscularly, and maintained with an intravenous (iv) infusion of phenobarbital 1 mg/ml, pancuronium 0.032 mg/ml, and morphine 0.06 mg·ml^-1 ^at a rate of 8 ml·kg^-1^·h^-1^. After a bolus injection of fentanyl 10 μ.kg^-1 ^iv a tracheotomy was performed and the lungs were ventilated through a shortened 8 mm inner diameter endotracheal tube (Mallinckrodt, Athlone, Ireland) in a volume-controlled mode (Servo i ventilator, Maquet, Solna, Sweden) with a Vt 6 ml/kg, a PEEP 5 cmH_2_O, and respiratory rate titrated to obtain normocapnea (35 < partial pressure of carbon dioxide (pCO_2_) < 45 mmHg). Lung injury was induced by repeated broncho-alveolar lavage with instillation of approximately 25 ml/kg warm saline solution per lavage. The end-point of the lavage was a sustained reduction in the partial pressure of oxygen (pO_2_)/fraction of inspired oxygen (FiO_2_) less than 100 mmHg during a period of 60 minutes.

### Measurements

Systemic and pulmonary arterial pressures, heart rate, mixed venous saturation, and body temperature were continuously monitored (CCombo 7.5-Fr, Edwards Life Sciences LLC, Irvine, CA, USA). Arterial blood gases were sampled every hour to measure partial pressure of arterial oxygen (PaO_2_), partial pressure of arterial carbon dioxide (PaCO_2_), pH and oxygen saturation (SpO_2_; ABL 500, Radiometer, Copenhagen, Denmark).

FOT was applied by using a system that has been described elsewhere [9]. Briefly, low amplitude sinusoidal pressure oscillations (about 1.5 cmH_2_O peak-to-peak) at 5 Hz were generated by a loudspeaker connected to the inspiratory line of the mechanical ventilator. Flow at the airway opening (Vao) was measured by a differential pressure transducer (PXLA02X5DN, Sensym, Milpitas, CA, USA) connected to a mesh-type heated pneumotachograph. Tracheal pressure was measured at the tip of the endotracheal tube by a differential pressure transducer (PXLA0075DN, Sensym, Milpitas, CA, USA). Signals were sampled at 200 Hz by the same A/D-D/A board used to control the loudspeaker and recorded on a personal computer.

### Experimental protocol

This study was the second part of a two-study protocol, designed to spare animals. Part 1 included a stepwise RM and computed tomography (CT)-scanning. For this reason, the ventilation trial started about five hours after the induction of lung injury.

The animals were randomized into two groups. One was treated with optimal PEEP (PEEPol) according to Xrs (FOT group), the other was treated with PEEP adjusted according to the ARDSNet protocol [1] (ARDSNet group). All animals underwent identical treatment before randomization, and there were no significant differences between the groups with regards to PaO_2_/FiO_2_, PaCO_2_, dynamic compliance (Cdyn), mean arterial pressure (MAP), and mean pulmonary arterial pressure (MPAP) before the intervention trial.

The duration of the protocol was 12 hours, with every experimental session involving two animals, one from each group, studied in parallel with a time shift of one hour to avoid the overlap of RM performed by the researchers. In both groups RMs were performed every two hours by increasing PEEP to 20 cmH_2_O for two minutes, preceded by tracheal suctioning for five seconds, to simulate a clinical situation in which a RM is performed to counteract derecruitment due to suctioning. Arterial blood gases were sampled and recorded five minutes after RMs and hourly. PEEP was adjusted after the RM every two hours in both groups.

**Figure 1:**
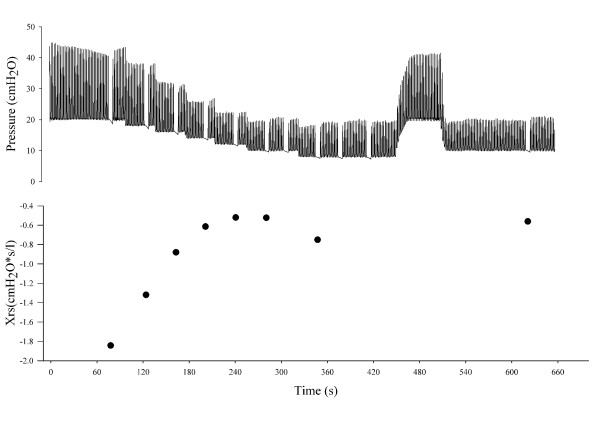
**PEEP optimization procedure according to optimal Xrs**. The upper panel shows tracheal pressure and the lower panel shows respiratory system reactance (Xrs) measured at end-expiration over time during a representative positive end-expiratory pressure (PEEP) optimization procedure. PEEP was increased up to 20 cmH_2_O, and then decreased in one-minute steps of 2 cmH_2_O while Xrs was continuously monitored. When Xrs started to decrease, PEEP was increased back to 20 cmH_2_O and finally set to the PEEP level corresponding to the maximum Xrs.

In the FOT group, PEEPol according to Xrs was identified as shown in Figure 1. Briefly, a decremental PEEP trial was performed immediately after the RM by a stepwise reduction of PEEP from 20 cmH_2_O in one minute-steps of 2 cmH_2_O until Xrs reached its maximum and started to decrease. PEEPol was defined as the PEEP level at the step preceding the first reduction of Xrs. Immediately after obtaining PEEPol, PEEP was increased again up to 20 cmH_2_O for one minute in order to restore lung volume and then it was brought back to PEEPol, which was maintained for the next two hours until the next scheduled optimization procedure.

In the ARDSNet group the optimization has been performed by following ARDSNet indications [1]. Moreover, in the ARDSNet group, PEEP was also adjusted between RMs whenever indicated.

By using this protocol, both groups (ARDSNet and FOT) received the same amount of RMs.

Respiratory rate and FiO_2 _were adjusted according to the ARDSNet protocol in both groups.

Leaks from the tracheal tube and ventilator circuits were continuously monitored for all the duration of the study.

At the end of the experiment, the animals were sacrificed by iv injections of potassium chloride (KCl). Thoracotomy was performed, and sections from the left lung (the lingula and the left lower lobe, two sections from each lobe) were fixed in buffered formalin solution and subsequently embedded in paraffin, sectioned at a thickness of 6 μm, and stained with H&E.

### Data analysis

#### Histopathology

The histopathological analysis was performed by a pathologist who was blinded to the outcome of randomization. Four fields for each pig (two from the lingula and two from the left lower lobe) were evaluated randomly. A grading scale (0 to 4) for four different histopathological markers of lung injury was used: presence of alveolar edema, hyaline membranes, inflammatory cells in alveoli, and inflammatory cells in septa, respectively (modified from [11]). Alveolar edema and hyaline membranes were graded according to the following criteria: 0-none, 1-focal in one to two fields, 2-focal in three to four fields, 3-widespread, 4-whole lung. Inflammatory cells in alveoli and inflammatory cells in septa were graded according to the following criteria: 0-none, 1-focal, a few cells, 2-widespread, a few cells, 3-all alveoli/septa, few cells, 4-brisk in all alveoli and septa. The evaluation scores for these markers were averaged to obtain a cumulative histopathology score for each animal.

#### Lung mechanics

The estimation of total respiratory system impedance (Zrs) was obtained from the flow and pressure signals by a least squares algorithm [12]. Zrs was expressed as real part, respiratory system resistance (Rrs), and imaginary part, respiratory reactance (Xrs).

#### Comparison between groups

The behavior of the two groups along the ventilation trial was compared in terms of ventilatory parameters (PEEP, Cdyn, plateau pressure (Pplat), and driving pressure (ΔP)), gas exchange (PaO_2_/FIO_2 _and PaCO_2_), and hemodynamics (MAP and MPAP). Cdyn values were provided by the ventilator using multiple regression analysis. The time of each measurement was referred to the first optimization procedure performed on the animal (time 0).

#### Statistical analysis

Data are expressed as mean (standard deviation). After testing normality by the Kolmogorov-Smirnov test, significance of differences between baseline parameters in the two groups was tested by unpaired t-test, when normality test succeeded, and by Mann-Whitney test, when normality test failed. Significance of differences between the two groups was tested by two-way analysis of variance (ANOVA) for repeated measurements using group and protocol step as factors. Multiple comparison after ANOVA was performed using Holm-Sidak test. Significance of differences between the histopathological scores given to the two groups was tested by Mann-Whitney test. Differences were considered statistically significant for *P *< 0.05.

## Results

The protocol could be followed without interruption in both groups, and it was possible to identify an optimal PEEP value after each RM in the FOT group. During RMs, moderate decreases in MAP and increases in MPAP were observed.

The experimental tracings recorded during a representative optimization procedure are reported in Figure 1. The pressure tracing shows the breathing cycles, the stepwise reduction of PEEP, and the end-expiratory pauses performed in order to establish the values of Xrs at end-expiration. The values of Xrs measured during the pauses are reported in the lower panel, where the expected increasing-decreasing pattern is evident. An optimal PEEP of 12 cmH_2_O was identified during this procedure, with a maximum Xrs value of -0.52 cmH_2_O*s/l.

**Figure 2:**
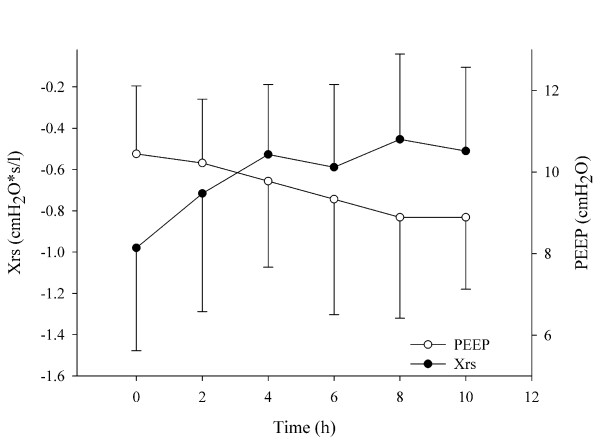
**Time course of PEEP and Xrs in the FOT group**. Mean and standard deviations of maximum respiratory system reactance (Xrs) values (closed symbols) and optimized positive end-expiratory pressure (PEEP; open symbols) assessed during the optimization procedure performed every two hours in the FOT group. In average, there was an improvement of oscillatory mechanics, which resulted in a reduction of optimal PEEP with time.

Figure 2 shows the values of the maximal Xrs and the optimal PEEP identified in all animals at the different optimization steps. The increase in Xrs clearly shows that there was an average improvement in the oscillatory mechanics with time, and this led to a progressively lower PEEP applied to the FOT group.

**Figure 3:**
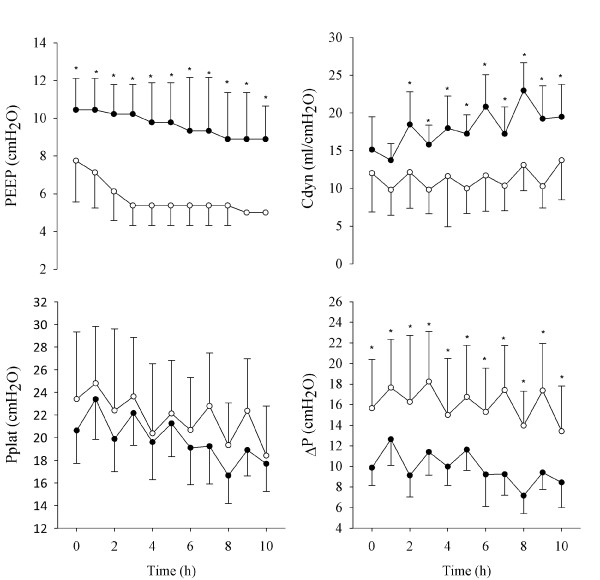
**Ventilatory and respiratory mechanics parameters over time**. Positive end-expiratory pressure (PEEP), plateau pressure (Pplat), driving pressure (ΔP), and dynamic compliance (Cdyn) for the forced oscillation technique (FOT) group (closed symbols) and for the acute respiratory distress syndrome (ARDS)Net group (open symbols). Data are presented as mean ± standard deviation. Significance of differences between the two groups at any protocol step are also reported. *, *P *< 0.01; +, *P *< 0.05.

**Figure 4:**
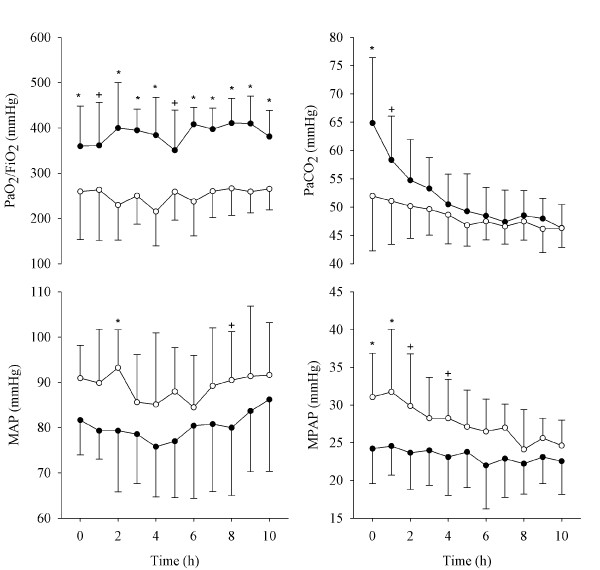
**Blood gases and hemodynamic parameters over time**. Partial pressure of arterial oxygen (PaO_2_)/fraction of inspired oxygen (FiO_2_), partial pressure of arterial carbon dioxide (PaCO_2_), mean arterial pressure (MAP), and mean pulmonary arterial pressure (MPAP) for forced oscillation technique (FOT) group (closed symbols) and for acute respiratory distress syndrome (ARDS)Net group (open symbols). Data are presented as mean ± standard deviation. Significance of differences between the two groups at any protocol step are also reported. *, *P *< 0.01; +, *P *< 0.05.

The relevant parameters measured every hour during the ventilation trial were averaged for all animals. The values of PEEP, Cdyn, Pplat, and ΔP are reported in Figure 3, and the values related to gas exchange and hemodynamics are reported in Figure 4. Cdyn, Pplat, and ΔP present an oscillatory pattern, likely due to the fact that the data were recorded every hour, while RMs and PEEP optimizations were performed every second hour. These data suggest that one hour after the PEEP optimization, the mechanical conditions of the lung were not as good as immediately after RM.

At the beginning of the trial, the optimization based on Xrs resulted in a significantly higher PEEP compared with that set in the ARDSNet group. These settings led to a significantly lower ΔP, a better oxygenation, and lower MPAP and MAP in the FOT group.

Over the course of the 12-hour experiment, PEEP decreased in both groups-from 10.4 (1.7) to 8.9 (1.8) cmH_2_O in the FOT group, and from 7.4 (2.1) to 5.0 (0) cmH_2_O in the ARDSNet group. These higher values of PEEP in the FOT group were associated with improved respiratory mechanics, as indicated by the significantly lower ΔP (decreasing from 9.88 (1.78) to 10.1 (2.05) compared with from 16.9 (5.2) to 13.4 (4.4) in the ARDSNet group) and the higher Cdyn for most of the course of the experiment (15.1 (4.4) to 15.7 (4.5) compared with 10.8 (4.2) to 13.7 (5.3) ml/cmH_2_O, respectively). There was a trend for lower Pplat in the FOT group, but the differences between the groups were not significant. At the end of the experiment, only changes in oxygenation and PEEP were still significantly different.

**Figure 5:**
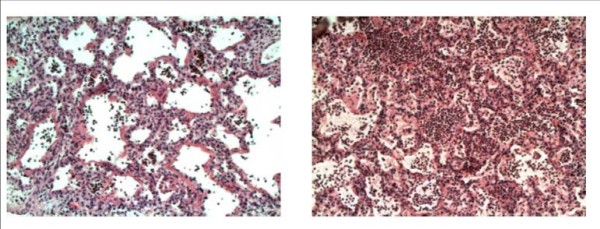
**Representative tissue samples**. Representative histopathology images of lung samples from the forced oscillation technique (FOT) group (right) and the acute respiratory distress syndrome (ARDS)Net group (left). There is more alveolar edema and inflammatory cells in the septa as well as in the alveoli in the animal ventilated with the positive end-expiratory pressure (PEEP) suggested by ARDSNet.

Qualitative and semi-quantitative analysis of histopathologic sections showed significant differences between the groups, as displayed in Table 1. Inflammatory exudation with hyaline membranes and signs of massive acute inflammation were found in both groups, but with a lower injury score in the FOT group. This is illustrated in Figure 5, with representative sections from both groups.

**Table 1:** Histopathological analysis

	ARSDNet group	FOT group	*P*
Alveolar edema	0.88 ± 0.92	0.06 ± 0.18	0.03
Hyaline membrane	0.94 ± 0.73	0.69 ± 0.70	0.50
Alveolar infl. Cells	1.31 ± 0.80	0.81 ± 0.46	0.15
Septal infl. Cells	2.69 ± 0.80	2.25 ± 0.65	0.25
**mean ± SD**	**1.45 ± 0.47**	**0.95 ± 0.37**	**0.03**

Lung injury scores for the forced oscillation technique (FOT) and the acute respiratory distress syndrome (ARDS)Net groups. Data are reported as mean ± standard deviation (SD).

ARDSNet group, group of animals in which PEEP was adjusted using the ARDSNet protocol; FOT group, group in which positive end-expiratory pressure (PEEP) was adjusted according to FOT measurement; infl., inflammatory.

## Discussion

The main result of this study was that during a 12-hour ventilation trial, the optimization of PEEP according to Xrs resulted in improved lung mechanics (assessed by conventional methods), a greater PaO_2_/FiO_2 _ratio and a reduced histopathologic evidence of VILI. The PEEP optimization procedure based on Xrs that we used in this study requires a RM followed by a decremental PEEP trial to identify PEEPol. The ARDSNet group was thus ventilated according to the ARDSNet protocol, with the addition of RMs performed at two-hour intervals to allow comparison between the two different PEEP strategies with all other interventions being equal. Previous animal studies have usually focused on short-term changes. To our knowledge, this is the first study to follow lung mechanics and ventilation parameters throughout the course of 12 hours, which more closely resembles a clinical situation with time enough for the more subtle mechanisms of VILI to have effect.

Even if the gold standard to assess lung volume recruitment is still CT scanning, there is increasing evidence that lung mechanics is a better surrogate than gas exchange variations for the assessment of lung recruitment at the bedside [13]. Starting from the pioneering work of Suter et al. [14], several studies suggested that the use of dynamic compliance [7,8,14,15] may guide in the identification of the optimal PEEP. A recent study in ALI/ARDS patients used a combination of oxygenation data (venous admixture) and lung mechanics obtained by electrical impedance tomography [16]. They reported that volume-dependent compliance seemed to be superior to dynamic compliance over the whole breath for monitoring lung recruitment and defining optimal PEEP. However, this method is labor intensive and expensive. Moreover, we have recently demonstrated that the volume-dependent component of compliance can only partially account for the non-linear behavior of the respiratory system during mechanical ventilation for ALI [10]. Also, the monitoring of esophageal pressure in order to maintain positive trans-pulmonary pressure has recently been suggested for PEEP optimization [17]. However, the necessity of an appropriate positioning of the esophageal balloon and the intrinsic difficulties in such a measurement implicate problems with the implementation of this technique in clinical applications.

Conversely, utilizing FOT, the peripheral lung mechanics can be continuously monitored via the ventilator circuit, and this could therefore be a preferable technique. Bellardine et al. applied FOT using the enhanced ventilation waveform approach on an animal model of ARDS to study changes in lung mechanics at different PEEP levels [18]. The authors found that optimal PEEP identified by CT scans minimizes mechanical heterogeneity, defined as the frequency dependence of Rrs and low-frequency elastance. However, this approach requires the assessment of mechanical impedances on a frequency range of 0.2 to 8 Hz and, therefore, is not suitable for patients with spontaneous breathing activity. In two previous studies we have shown that single frequency FOT at 5 Hz can be used to accurately evaluate lung volume de-recruitment overcoming several limitations of Cdyn, such as the effects of non-linearities in the respiratory system and the need for deep sedation or paralysis of the patients [9,10]. The results of these studies also suggested that by monitoring Xrs it is possible to continuously assess the development of lung collapse and to evaluate the efficacy of RMs, allowing bedside characterization of lung recruitability. In the present study, we implemented these findings in designing a strict protocol based on PEEP optimization according to Xrs performed every two hours. What we found is that optimal PEEP set on the basis of Xrs changes was clearly advantageous compared with PEEP settings according to the ARDSNet protocol, which only uses oxygenation data.

However, in the FOT group in which Xrs was continuously monitored, we occasionally observed decreases in Xrs during the two-hour intervals between the scheduled RMs, but no adjustments were made. Thus we did not fully use the information provided by FOT. An improved clinical protocol could perhaps be developed, including RMs coupled with Xrs monitoring for PEEP optimization, with the addition of using Xrs triggers for performing subsequent RMs immediately when de-recruitment occurs.

Hemodynamically, there were no differences between the FOT and ARDSNet groups except for the pulmonary artery pressure. The lower pulmonary arterial pressure in the FOT group could be due to successful lung recruitment-the optimal PEEP keeping the lung open and thus decreasing pulmonary vascular resistance. During the latter half of the experimental period, this difference was no longer significant. This may have been due to the long duration of the experiment, with attenuation of the lung injury in both groups explained by the recovery of the lung often seen in the lavage model.

### Limitations of the study

We performed histopathologic analysis of several sections of lung tissue after sacrifice. We chose not to excise whole lungs, precluding true quantitative analysis of histopathologic changes, but the qualitative and semi-quantitative multi-parameter score based on several previous studies showed clear and significant differences between the groups.

The saline lavage model of lung injury causes surfactant depletion and atelectasis that is easily recruitable, in contrast to the heterogeneous inflammatory changes of long-lasting nature that characterize the human ARDS. This could explain the relatively low ventilatory pressures and PEEP settings that adherence to the ARDSNet protocol dictated in the present study. It could also account for the clinical improvement through the course of the experiment, including both blood gases and ventilatory settings. An advantage of this was that the final damage seen in the histopathologic sections could most likely be attributed to mechanical ventilation-the focus of the study-rather than the initial lavage injury.

In this study we compared PEEP optimization performed by FOT with the one based on oxygenation data as suggested by the ARDSNet. With this experimental protocol, we could not compare our results with the ones that would have been obtained by using other optimization procedures based on the assessment of mechanical properties (such as Cdyn). However, in a previous study we have shown that PEEPol defined by Cdyn is similar but not equal to the one identified by FOT. Moreover, given that FOT is not affected by the non-linearities of the respiratory system, nor by the spontaneous breathing of the patient, and it can be easily integrated in mechanical ventilators, we think that single-frequency FOT could be easier than other techniques to be applied in clinical practice.

Finally, in the present study the esophageal pressure was not measured, and thus changes in Xrs include both changes in lung and chest wall mechanics. However, we have previously shown, by using mathematical models, that the contribution of the changes in chest wall compliance to Xrs is negligible compared with the contribution of lung volume recruitment/derecruitment and, therefore, it does not affect the estimation of PEEPol [10].

## Conclusions

The results indicate that there is a scientific basis for implementing an open-lung strategy that includes RMs and PEEP optimization using FOT. Future studies should aim to confirm these observations in ALI/ARDS patients, possibly taking the protocol one step further by utilizing the continuous monitoring of reactance and investigating the feasibility of a reactance based trigger for RMs. Considering that the optimization procedure based on Xrs can be easily integrated in commercial mechanical ventilators and that it provides continuous monitoring of the mechanical properties of the peripheral airways, we conclude that FOT could be an important component in a strategy for delivering protective ventilation to patients with ALI.

## Key messages

• In a surfactant-depletion model of ALI, during a decremental PEEP trial following a RM there was always a PEEP level at which the respiratory system reactance measured by FOT reached a maximum.

• When PEEP was set to the value that maximized the reactance, higher PEEP levels, improved lung mechanics, and better oxygenation were observed compared with those measured when PEEP was set following standard clinical protocols based on oxygenation (ARDSNet).

• A PEEP setting strategy based on the optimization of respiratory reactance produced less histologic signs of lung injury compared with the oxygenation-based ARDSNet protocol after a 12-hour ventilation trial.

## Abbreviations

ALI: acute lung injury; ANOVA: analysis of variance; ARDS: acute respiratory distress syndrome; Cdyn: dynamic compliance; CT: computed tomography; FiO_2_: fraction of inspired oxygen; FOT: forced oscillation technique; H&E: hematoxylin and eosin; MAP: mean arterial pressure; MPAP: mean pulmonary arterial pressure; PaCO_2_: partial pressure of arterial carbon dioxide; PaO_2_: partial pressure of arterial oxygen; pCO_2_: partial pressure of carbon dioxide; PEEP: positive end-expiratory pressure; PEEPol: open lung PEEP; Pplat: plateau pressure; pO_2_: partial pressure of oxygen; RM: recruitment maneuver; Rrs: respiratory system resistance; SpO2: oxygen saturation; VILI: ventilator-induced lung injury; Vt: tidal volume; Xrs: respiratory system reactance; Zrs: total respiratory system impedance; ΔP: driving pressure.

## Competing interests

Politecnico di Milano University, the institution of EZ, PP, AP and RD, owns a pending patent on the detection of lung recruitment by FOT, which to date has not been licensed to any company.

## Authors' contributions

PK contributed to the study design, participated in the experimental activity and drafting the manuscript. EZ contributed to the study design, participated in the experimental activity, performed the data processing, and contributed to the data interpretation and drafting the manuscript. MAO participated in the experimental activity. PP designed the experimental set-up, participated in the experimental activity, and contributed to data processing. GH contributed to the study design, and critically revised the manuscript. AP contributed to the study design. AL critically revised the manuscript. PF contributed to the study design, participated in the experimental activity, and in the interpretation of the results and contributed to drafting the manuscript. RD contributed to the study design, designed the experimental set-up, participated in the experimental activity, and in the interpretation of the results and contributed to drafting the manuscript.
